# Change in Methicillin-Resistant *Staphylococcus aureus* Testing in the Intensive Care Unit as an Antimicrobial Stewardship Initiative

**DOI:** 10.31486/toj.22.0103

**Published:** 2023

**Authors:** Hayden L. Smith, Samuel P. DuMontier, Amanda M. Bushman, Jonathan R. Hurdelbrink, William J. Yost, Steven R. Craig

**Affiliations:** ^1^ Medical Education Services, UnityPoint Health–Des Moines, Des Moines, IA; ^2^ Department of Pharmacy, UnityPoint Health–Des Moines, Des Moines, IA; ^3^ Department of Health Sciences, Drake University, Des Moines, IA

**Keywords:** *Anti-infective agents*, *diagnosis*, *diagnostic techniques and procedures*, *intensive care units*, *methicillin-resistant* Staphylococcus aureus, Staphylococcus aureus, *vancomycin*

## Abstract

**Background:** Methicillin-resistant *Staphylococcus aureus* (MRSA)–associated infections are a cause of morbidity and mortality in the intensive care unit (ICU). Vancomycin is a treatment option but is not without risks.

**Methods:** A MRSA testing change—the switch from culture to polymerase chain reaction—was implemented at 2 adult (tertiary and community) ICUs located in a Midwestern US health system. Data from 2016 to 2020 were included in the study, and the median change in time to test results was examined.

**Results:** During the study period, 71% of 19,975 patients seen at the 2 ICUs received MRSA testing. In the preintervention period, 91% and 99% of patients at the tertiary and community hospitals received testing via culture, respectively. Culture testing was used 1% and ∼0% of the time at the tertiary and community hospitals, respectively, in the postintervention period. A counterfactual estimate showed 36 (95% credible interval [CrI], 35, 37) and 32 (95% CrI, 31, 33) fewer hours until results were available at the tertiary and community hospitals, respectively.

**Conclusion:** After the testing change, MRSA results were available in less time. Obtaining results sooner can assist with antimicrobial stewardship through the potential delay in initiating therapies such as vancomycin and/or quicker de-escalation of such therapies.

## INTRODUCTION

Methicillin-resistant *Staphylococcus aureus* (MRSA) infection is a leading cause of severe morbidity, mortality, and economic burden on patients and health care systems.^1^ MRSA-colonized patients are commonly present in the intensive care unit (ICU). Between 1992 and 2004, >60% of ICU *S aureus* infections were attributed to MRSA in the United States and Canada.^2^ Between 1999 and 2005, MRSA-related hospitalizations more than doubled, resulting in increasing awareness and need for preventive measures and effective treatment.^1^ The Centers for Disease Control and Prevention estimated 80,461 invasive MRSA infections occurred in the United States in 2011.^3^

Methicillin resistance among *S aureus* isolates was first identified in the early 1960s.^4^ MRSA is a common pathogen that not only is part of our own microbiota but also can cause skin, soft tissue, bloodstream, bone, heart, and respiratory infections.^5,6^ To reduce critical illness, the Infectious Diseases Society of America recommends initiation of empiric antibiotics if clinical suspicion is high for MRSA infection.^7,8^

Vancomycin is often the first choice for empiric antibiotic therapy because of its coverage of MRSA, but vancomycin therapy has risks that can disrupt patient care. Acute kidney injury is one of the most significant adverse effects of vancomycin. Patients in the ICU are often susceptible to acute kidney injury given their comorbidities and the numerous potentially nephrotoxic agents used in critical care treatment, such as vasopressors, intravenous contrast dye, diuretics, certain beta-lactam antibiotics, and aminoglycosides.^9-11^ Duration of vancomycin exposure, dose amount, and specific patient vulnerabilities (eg, previous chronic kidney disease, elevated body mass index, severity of illness, and hemodynamic support) can contribute to nephrotoxicity. The consequences of nephrotoxicity include prolonged hospital stay, potential need for dialysis, and increased risk of mortality.^9,12^ Antibiotic regimens must be tailored, not only to deter antibiotic resistance but also to limit potential patient harm from unnecessary antibiotic exposure, and antimicrobial stewardship in MRSA pharmacotherapy is a rapidly developing practice.

Early detection of MRSA can help guide and treat acute infection. MRSA polymerase chain reaction (PCR) nasopharyngeal swab testing is a rapid detection test that has shown excellent performance characteristics, especially in the detection of pneumonia.^13^ Because MRSA PCR nares screening is an effective rapid detection test, it also has the potential to reduce vancomycin use.^14-16^

The objective of this study was to decrease the time between the ordering of a MRSA test and the availability of test results for patients admitted to the adult ICU.

## METHODS

### Study Design and Variables

We initiated a multisite intervention for patients admitted to 2 adult ICUs in the same health system: a tertiary hospital (Level 1 trauma center with 37 ICU beds) and a community hospital (Level IV trauma center with 15 ICU beds) located within 3 miles (Manhattan distance) of each other in the same Midwestern city in the United States.

On February 1, 2018, the default MRSA test in the laboratories serving these facilities was changed from culture to PCR for patients being admitted to the ICU. The purpose of the change was to make MRSA test results available to clinicians sooner. For testing, the tertiary hospital used an in-house laboratory, and the community hospital used an offsite laboratory. For the first 3.5 years of the study period, the community hospital used the tertiary hospital laboratory. For the remainder of the study period, the community hospital used a newly built offsite laboratory within the health system.

The study received institutional review board approval (IM2017-100). Study data were collected from January 1, 2016, through October 31, 2020, a time range that includes 25 months of preintervention data and 33 months of postintervention data. We selected this time range to show that test results were stationary across time and that results were not perceptibly impacted after the community hospital switched laboratories.

Testing consisted of either a nasopharyngeal swab with culture grown on agar medium (Becton, Dickinson and Company; laboratory time of 48 hours) or a nasopharyngeal swab with PCR testing using the GeneXpert System (Cepheid; laboratory time of 70 minutes). Both tests were available throughout the entire study period, but PCR was the default/preferred method during the postintervention period. No additional methodological changes were implemented. Test results were uploaded to the laboratory tab in the electronic health record, but no automated notification was sent to the ordering provider when the results were uploaded. The cost of the culture test was $8 for the hospital ($71 for the patient), and the cost of the PCR test was $14 for the hospital ($137 for the patient).

Collected data were hospital indicator (tertiary or community), date and time of admission, date and time of ordered test, test type (culture or PCR), test result (positive or negative), test result date and time, and whether the patient was prescribed vancomycin prior to the posting of the MRSA test result in the medical record (yes or no). An intervention period indicator was constructed (preintervention or postintervention period). Patient demographic data collected were age and sex.

### Data Analysis

Continuous data are reported as medians with interquartile ranges (IQR) and categorical data as counts with percentages. A Bayesian quantile regression model was fit to data to examine the level change in time to laboratory results at each hospital. Model details are provided in the Appendix. Results are presented as estimated median differences between time of test ordered and laboratory result posted for the preintervention and postintervention periods. A counterfactual estimate of change in time to receipt of the laboratory results at the midpoint of the postintervention period was constructed (ie, contrasting (E(Y^X=0^)) and (E(Y=*y* | do(X=*x*))) with Y = time until results, X = laboratory change [0 represents no laboratory change, and 1 represents laboratory change] and assuming weak ignorability [X || Y^x=0^ and X || Y^x=1^] and a well-defined intervention). This process controlled for changes in time until results were uploaded (ie, model slopes) within and across study periods. All model-based estimates are reported with 95% credible intervals (CrI), and additional details are provided in the Appendix.

A Monte Carlo (MC) simulation model was fit to quantify the percentage of patients in the postintervention period at the tertiary hospital who would probabilistically test positive for MRSA. This estimate represents the hypothetical percentage of positive patients for whom treatment could have been delayed if vancomycin were not initiated until after MRSA results were known. This calculation was based on the empiric number of positive tests in patients receiving vancomycin before the MRSA results were known during the postintervention period at the tertiary hospital using a beta distribution with 50,000 MC samples.

## RESULTS

### Study Sample

During the 58-month study period, 71% (n=14,152) of the 19,975 adult ICU patients received MRSA testing. These patients were distributed with 4,347 and 1,607 in the preintervention period and 6,156 and 2,042 in the postintervention period at the tertiary and community hospitals, respectively. Patients at the tertiary center were 78% male with a median age of 64 (IQR, 52, 74) years, while the community hospital patients were 70% male with a median age of 62 (IQR, 48, 75) years.

### MRSA Testing

During the preintervention period, 91.8% (3,989/4,347) and 98.8% (1,588/1,607) of patients at the tertiary and community hospitals, respectively, received MRSA testing via culture. Use of culture testing was 0.8% (49/6,156) and ∼0% (2/2,042) at the tertiary and community hospitals, respectively, during the postintervention period. Across the study periods, the overall MRSA positivity rate was 9.9% (1,399/14,144; missing 8), represented by 8.9% (387/4,346; missing 1) and 9.8% (158/1,607) during the preintervention period and 10.2% (627/6,150; missing 6) and 11.1% (227/2,041; missing 1) during the postintervention period at the tertiary and community hospitals, respectively. The median time between all reviewed MRSA tests in the admitted patients for the ICUs was constant across time, with the tertiary hospital being 2.5 hours and the community hospital being 8.0 hours for both study periods.

### Time to Test Results

Sixteen (0.1%) of the 14,152 reviewed MRSA tests were considered outliers and removed from the analytic models because the time to receiving results was >4 days. Estimated median times to receipt of results during the preintervention and postintervention periods by hospital are reported in the Table and visualized in the Figure. Model results revealed an estimated median difference in time to receipt of test results of 38.2 fewer hours (95% CrI, 37.9, 38.5) at the tertiary hospital and 28.8 fewer hours (95% CrI, 28.0, 29.6) at the community hospital. The dispersion (width of the IQRs) for time to receipt of test results in the preintervention period at the tertiary and community hospitals was 13 and 12 hours, respectively, vs 1 and 7 hours in the postintervention period. The counterfactual median estimate of difference in time to receipt of test results at the midpoint of the postintervention period was 36.0 fewer hours (95% CrI, 35.4, 36.6) at the tertiary hospital and 31.6 fewer hours (95% CrI, 30.5, 32.8) at the community hospital. Posterior density plots for model estimates are provided in the Appendix.

**Table.** Time to Receiving Methicillin-Resistant *Staphylococcus aureus* (MRSA) Test Results for Patients Admitted to an Adult Intensive Care Unit, n=14,136^a^

	Time to MRSA Results, Hours, Median (Interquartile Range)	
Hospital	Preintervention	Postintervention
Tertiary	41.2 (95% CrI, 41.0, 41.5)	3.0 (95% CrI, 2.8, 3.1)
Community	41.7 (95% CrI, 41.2, 42.2)	12.9 (95% CrI, 12.2, 13.5)

^a^Sixteen patients (0.1%) were not included in the analyses because the time to receiving MRSA test results was >4 days.

CrI, credible interval.

**Figure.**
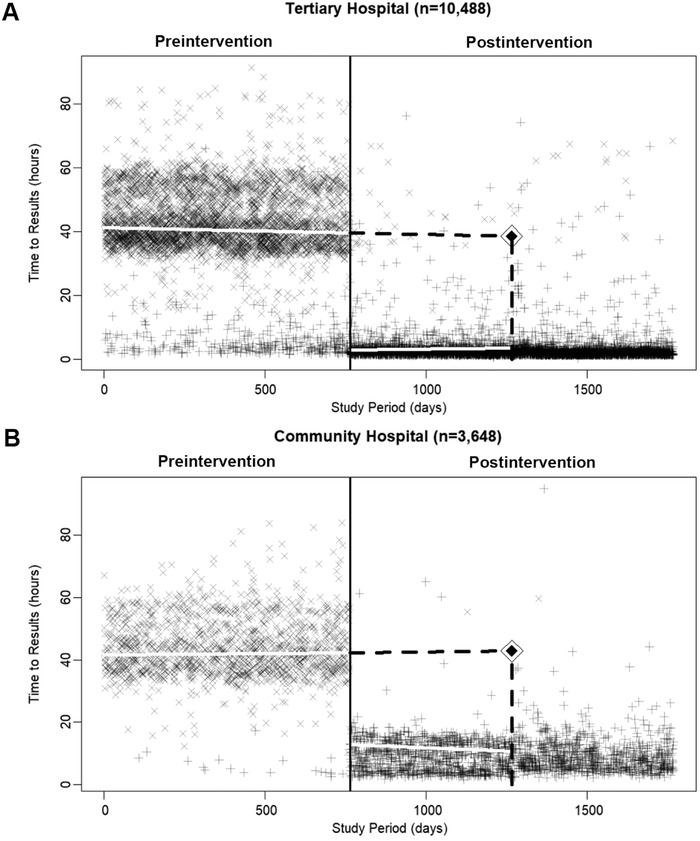
**Plotted time in hours until receipt of methicillin-resistant *Staphylococcus aureus* test results for patients admitted to 2 adult intensive care units by study period day. The top panel (A) presents tertiary hospital data, and the bottom panel (B) presents community hospital data. The solid black vertical line represents when the test change occurred; the solid white lines are the estimated time to receipt of test results per period; the dashed black lines represent the counterfactual estimate at the midpoint of the postintervention period with a diamond located at the estimate. In the bottom panel after the vertical dashed line is a slight possible reduction in time to receipt of test results at the community hospital when it changed laboratories. Sixteen patients (0.1%) were dropped from the analyses because the time to receiving MRSA test results was >4 days.** Symbols: x=culture test; +=polymerase chain reaction test.

### Vancomycin Administration

At the tertiary hospital, 19.3% (765/3,959; missing 1) of patients had a vancomycin dose initiated prior to receiving a negative MRSA test during the preintervention period vs 19.2% (1,062/5,523; missing 6) of patients during the postintervention period. At the community hospital, 12.1% (175/1,449) of patients had a vancomycin dose initiated prior to receiving a negative MRSA test during the preintervention period vs 16.0% (n=290/1,814; missing 1) during the postintervention period. Based on patient study data from the tertiary hospital during the postintervention period, if vancomycin were not initiated until test results were known, the simulation estimated that 16% (95% CI, 14%, 18%) of these patients may have tested positive and had delayed initiation of vancomycin.

## DISCUSSION

The focus of this study was to evaluate the time to receipt of MRSA results in admitted adult ICU patients after implementing a testing change. At the tertiary hospital that used an in-house laboratory, the median time to receipt of results decreased by 38 hours. At the community hospital that used an offsite laboratory, the time decreased by almost 30 hours. Secondary to this change, the percentage of positive tests increased during the postintervention period; however, speculation about whether this change was related to the testing modality or endemic changes in the population is outside the scope of this project. The variability in time to receipt of results decreased in the postintervention period at both hospitals compared to the time ranges in the preintervention period.

The patients for whom vancomycin was initiated prior to receiving MRSA test results represent patients who could benefit from not having any delays in MRSA testing information. Given a patient's presentation and pretest probability for MRSA infection, the use of empiric vancomycin may be necessitated in some patients but potentially delayed until PCR test results are known in other patients given the test's quick turnaround time. After the testing change, results were available sooner at both hospitals, which can result in earlier de-escalation or discontinuation of antibiotics in patients testing negative and potentially few vancomycin troughs. Both of these possible effects could represent cost savings and lessen the care burden for patients.

Multiple studies have indicated that a MRSA PCR nasopharyngeal swab test has a relatively high negative predictive value and is an effective approach to de-escalating or discontinuing MRSA antibiotic therapy—most notably in patients treated empirically for MRSA pneumonia.^13,15,16^ Moreover, Baby et al showed that early de-escalation of MRSA antimicrobial therapy did not lead to worse outcomes.^15^ In situations where MRSA prevalence or overall suspicion for infection is low, we can infer that avoiding empiric anti-MRSA therapy until MRSA nasal screening results are verified is plausible. In our study, we saw a nonappreciable change in the initiation of vancomycin in patients testing negative for MRSA, along with a slight increase in positivity rates. In theory, there may be an opportunity for a change in practice via a delayed initiation of empiric therapy in low-suspicion patients, although at the study hospitals, delayed initiation seemed to be an area of continued caution for clinicians. Future investigations of this topic may be of interest to researchers in understanding whether such antimicrobial stewardship practice is practical.

### Limitations and Considerations

The postintervention study period could have been impacted by coronavirus disease 2019. The respiratory pandemic led to an increase in mechanical ventilation use that puts patients at increased risk for a superimposed infection such as MRSA pneumonia. The increase in respiratory illness could have affected the data in ways that we are unable to quantify. Additionally, not all ICU patients during the postintervention period received a MRSA PCR screen per protocol.

We did not review the vancomycin courses for patients because initially prescribed vancomycin dosages can vary between patients based on provider preferences and patient presentation. For example, different dosages may have varying coverage and can be titrated to patients based on various factors (eg, kidney function). Additionally, MRSA test result notifications were not automatically sent to the ordering providers. This communication gap likely slowed the process of discontinuation of antibiotics and may have varied between providers. In other words, even though the MRSA results were posted in the medical record, the provider was not notified, and the patient may have remained on the initially prescribed antibiotic course until it was completed or until the provider saw and acted on the test results. Because of these issues, these data may not be generalizable to other institutions.

Some patients received MRSA testing via PCR during the preintervention period, while some patients received culture-based testing during the postintervention period. Noncompliance with the default testing method per study period could underestimate the optimal theoretical improvement in time to receipt of test results because the analyses are based on an intent-to-treat design. However, with the use of the 50th percentile in the quantile regression, the results should not have been overly influenced by a smaller or larger lag in accessibility of results to providers for a minority of patients (ie, patients being tested via PCR in the preintervention period or patients being tested via culture in the postintervention period). As shown in the Appendix, a deep artificial neural network model was fit to data to show what results from an adaptive nonlinear model could look like given increasing noncompliance at the end of the preintervention period.

Given the sequence design of the study, patients were not randomized to testing method. The lack of randomization could result in slight issues of patient exchangeability between study periods, although the study of a testing/laboratory change should occur in a standardized fashion and negate concerns about patient differences because the focus was on the testing process. Last, the study did not have a staggered rollout across the hospitals or include a negative control group to help understand the potential for an unknown historic bias occurring at the time of the testing change. This concern was indirectly addressed by providing an extended preintervention and postintervention time series that revealed no apparent occurrences of exogenous shocks or slope changes in the series beyond the study intervention point.

## CONCLUSION

The study revealed a decrease in time to receipt of MRSA test results after the testing method was changed and a decrease in the variability of these times in the postintervention period. The intervention was implemented at 2 types of facilities to show the potential generalizability of the change and to corroborate findings. Based on the study results, PCR nasopharyngeal swab can reasonably be inferred to be a valuable screening tool to identify MRSA respiratory infection.

## Figures and Tables

**Figure A1.**
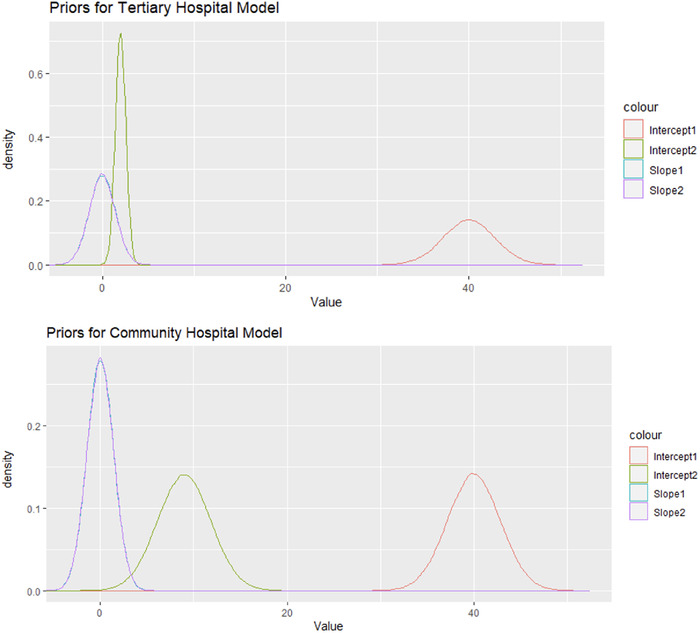
**The top panel presents the priors used in the tertiary hospital model, and the bottom panel presents the priors used in the community hospital model. Priors for slope values for the 2-line segments were the same and mostly overlap in the figures.** (For readers of the print publication, a color version of this figure is available at https://www.doi.org/10.31486/toj.22.0103.)

**Figure A2.**
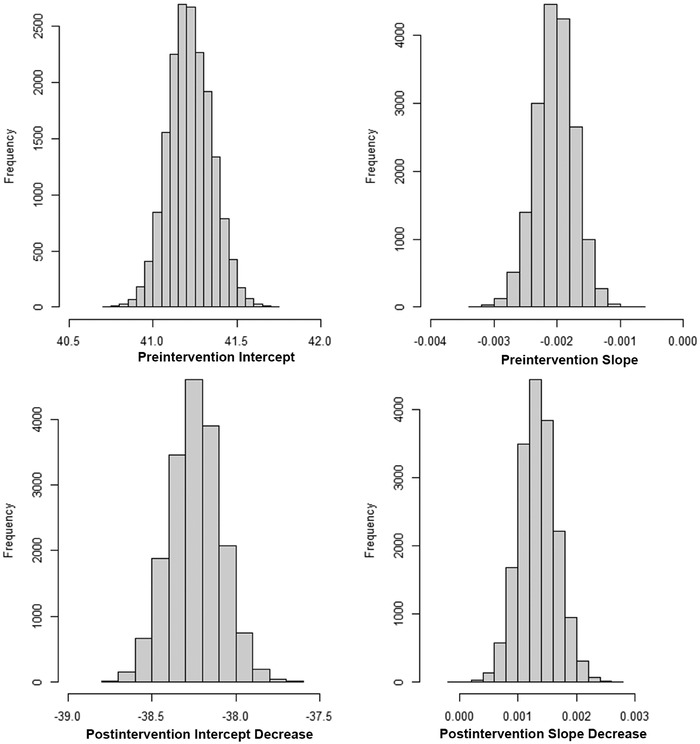
Tertiary hospital posterior estimates. The x-axes represent the Bayesian quantile regression model coefficients related to receipt of methicillin-resistant *Staphylococcus aureus* results. Preintervention is the period before the switch from culture testing to polymerase chain reaction testing. Slopes represent change in time (hours) to receipt of results per day increase during the designated period (ie, preintervention or postintervention).

**Figure A3.**
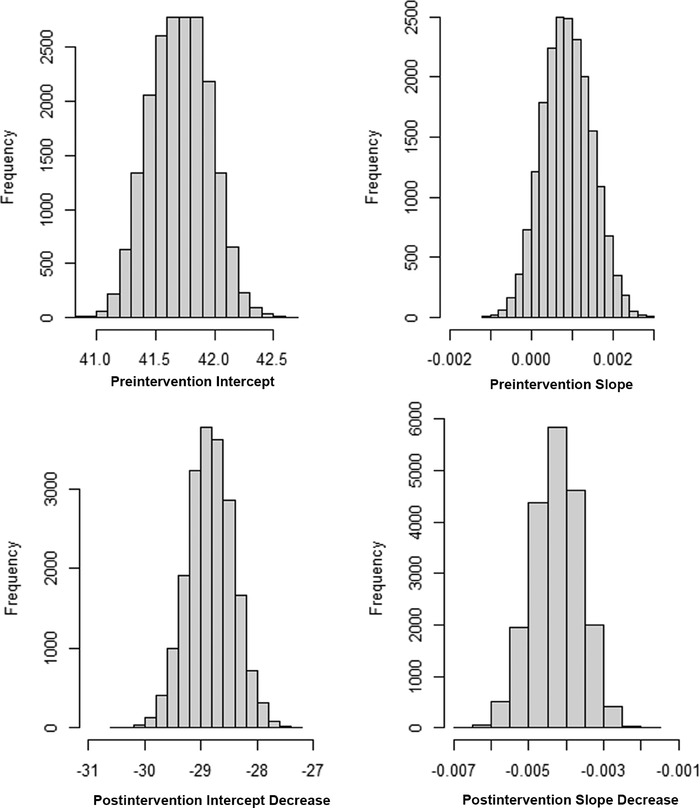
Community hospital posterior estimates. The x-axes represent the Bayesian quantile regression model coefficients related to receipt of methicillin-resistant *Staphylococcus aureus* results. Preintervention is the period before the switch from culture testing to polymerase chain reaction testing. Slopes represent change in time (hours) to receipt of results per day increase during the designated period (ie, preintervention or postintervention).

**Figure A4.**
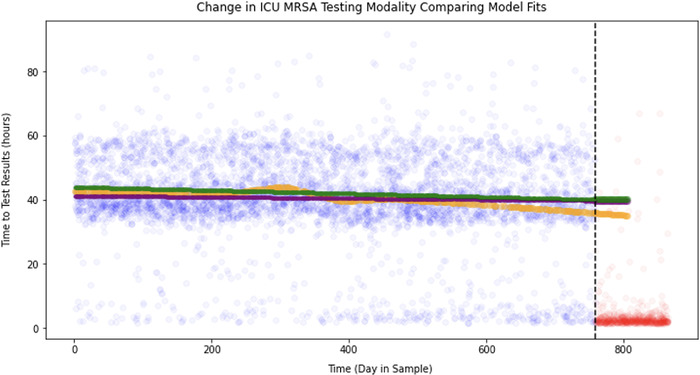
**Two alternative model fits. The green line represents the quantile regression from the paper. The purple line is a robust regression (ie, Huber), while the orange line is a deep artificial neural network (ie, 3 hidden layers with rectified linear units activations, initial null bias and Glorot normal weights, batch normalization, and 10% dropout). The neural network shows what a more adaptive nonlinear prediction can look like given the presence of increased noncompliance in default testing method near the end of the study period (ie, dashed vertical line).** ICU, intensive care unit; MRSA, methicillin-resistant *Staphylococcus aureus.* (For readers of the print publication, a color version of this figure is available at https://www.doi.org/10.31486/toj.22.0103.)

## Notes

*This article meets the Accreditation Council for Graduate Medical Education and the American Board of Medical Specialties Maintenance of Certification competencies for Patient Care, Medical Knowledge, and Practice-Based Learning and Improvement.*

## Appendix. Statistical Model for Change in Methicillin-Resistant *Staphylococcus aureus* Testing in the Intensive Care Unit as an Antimicrobial Stewardship Initiative

The a priori analytic plan was to examine data and fit a Bayesian interrupted time series and to report estimated medians and a counterfactual. The study model was conducted using runjags, version 2.2.0-2, in R, version 4.0.2 (R Foundation). Model chains for estimates were initiated based on the empiric median time to result values for the 2 study periods per hospital using study data and a random draw from the uniform distribution for sigma and rho (ie, sigma=runif(1, 0.1, 10); rho=runif(1, –1, 1)). Priors used in the models are presented below.

**Tertiary hospital:**

Preintervention intercept ∼ dnorm(40, 2)
Preintervention slope ∼ dnorm(0, 1)
Postintervention intercept ∼ dgamma(5, 2)
Postintervention slope ∼ dnorm(0, 1)
Sigma ∼ dunif(0.1, 10)
Rho ∼ dunif(–1, 1)

**Community hospital:**

Preintervention intercept ∼ dnorm(40, 2)
Preintervention slope ∼ dnorm(0, 1)
Postintervention intercept ∼ dnorm(8, 2)
Postintervention slope ∼ dnorm(0, 1)
Sigma ∼ dunif(0.1, 10)
Rho ∼ dunif(–1, 1)

An examination of the above model revealed patients in the preintervention and postintervention periods could have received the nondefault test (ie, polymerase chain reaction [PCR] in lieu of the culture test and vice versa). These noncompliant tests represented a more complex underlying data-generating process where the 2 tests were influencing the dependent variable estimates in the model in both periods. Varying use of the 2 tests was also seen in a multimodal residual distribution for the model errors. The existence of noncompliance also resulted in predictions of estimates varying based on what the previous test was for that prediction. For example, if the prior test was a culture with a 44-hour result time and the next test was a PCR with a 4-hour result time, predicted estimates would be a weighted value around 20 hours until results and not near the actual value of around 4 hours at the tertiary hospital. Also, if the next test was a culture, its predicted estimate was also a weighted version based now on the PCR test. The noncompliance resulted in a varying identification of the estimated outcome. Second, autocorrelation function and partial autocorrelation function plots revealed a lack of correlation patterns in model errors across time.

The purpose of the study was to model the median change in time to results between the study periods, so a Bayesian quantile regression model was selected to examine the primary objective of the study because it would be able to provide a robust estimate of the median. The bayesQR version 2.3 R package was used to fit study data in a quantile regression using an intercept, preintervention period slope, postintervention period indicator, and postintervention slope (interaction term) variable with 20,000 samples with a 100 burn-in, thinning=2, and chains=2. Priors for the model are presented in Figure A1. Of note, bayesQR uses asymmetric Laplace densities that restricted prior distribution options, which meant gamma priors could no longer be used.

**Tertiary hospital:**

Preintervention intercept ∼ dnorm(40, 2)
Preintervention slope ∼ dnorm(0, 1)
Postintervention intercept ∼ dnorm(3, 0.4)
Postintervention slope ∼ dnorm(0, 1)
Sigma ∼ default flat prior

**Community hospital:**

Preintervention intercept ∼ dnorm(40, 2)
Preintervention slope ∼ dnorm(0, 1)
Postintervention intercept ∼ dnorm(9, 2)
Postintervention slope ∼ dnorm(0, 1)
Sigma ∼ default flat prior

Posterior estimates from the Bayesian quantile regression model for the tertiary and community hospitals are presented in Figures A2 and A3, respectively. In addition, a sensitivity analysis represented by fitting 2 alternative models to the tertiary hospital data is visualized in Figure A4.
